# Impact of High-Intensity Circuit Resistance Exercise on Physical Fitness, Inflammation, and Immune Cells in Female Breast Cancer Survivors: A Randomized Control Trial

**DOI:** 10.3390/ijerph19095463

**Published:** 2022-04-29

**Authors:** Kwang-Jin Lee, Keun-Ok An

**Affiliations:** 1 Department of Physical Education, Chungbuk National University, Cheongju 28644, Korea; rhkdwls@cbnu.ac.kr; 2 Sports Medicine Major, Division of Sports, Korea National University of Transportation, Chungju 27469, Korea

**Keywords:** high-intensity circuit resistance exercise, physical fitness, inflammatory cell, immune cells, female breast cancer survivors

## Abstract

Questions remain about whether resistance exercise has a positive effect on immune and inflammatory cells. The purpose of this study was to evaluate the effect of 12 weeks of high-intensity circuit resistance exercise (HCRE) on inflammation and immune cells, and physical fitness, of female breast cancer survivors (FBCSs). Thirty FBCSs were randomly assigned to the HCRE (*n* = 15) and control (*n* = 15) groups. HRCE was administered for 50 min a day, 2–3 times a week, for 12 weeks. The control group only performed activities of daily living during the study period. Baseline and post-intervention measures included body composition, muscular strength, muscular endurance, flexibility, reaction time, balance, inflammation, and immune cell measurements. The results showed that HCRE improved body mass index, body fat, muscle mass, grip strength, back muscle strength, sit-up, whole-body reaction, standing on one leg with eyes closed, Y-balance test, and NKCA in FBCSs. The improvement of physical strength and immune cells of FBCSs was achieved using the 12-week HRCE program. Future studies must analyze various exercise intensities and types, and should be conducted on other cancer survivors. In addition, strategies should be developed to allow FBCSs to participate in resistance training.

## 1. Introduction

In 2020, there were approximately 2.2 million cases of breast cancer worldwide. Breast cancer accounts for one in four cancer cases and one in six cancer-related deaths in women, ranking first in the incidence rate of cancer among women in most countries [1]. Korea has a high cancer incidence rate, along with North America and Europe, with 59.8 breast cancer cases per 100,000 people [2]. Westernized diet, prolonged exposure to estrogen, and genetic factors are the main causes of breast cancer. The disease is most common among middle-aged women between the ages of 35 and 64 years, but the survival rate is reported to be higher than 90% [3,4].

Regular exercise, including aerobic exercise, resistance training, aqua aerobics, Tai Chi, and yoga, is recognized as an alternative treatment method for breast cancer because it minimizes the side effects of breast cancer treatment, increases physical strength, and improves the quality of life of female breast cancer survivors (FBCSs) [5]. Resistance exercise (RE) has a positive effect on improving upper and lower body strength, body composition, physical function, and quality of life in FBCSs [6,7], suggesting that regular participation in RE should be recommended as a key strategy for cancer recovery and risk reduction in FBCSs. However, most cancer survivors do not participate in RE [8]. In addition, questions remain about whether RE has a positive effect on immune and inflammatory cells. Hagstrom et al. [9] reported that RE performed at 80% intensity of one repetition maximum (RM) for 16 weeks decreased the expression of tumor necrosis factor-α by natural killer (NK) and natural killer T (NKT) cells in the exercise group, but there was no significant difference in NK cells (%) and NKT cells (%). Simonavice et al. [10], however, reported that RE performed at 80% intensity of one RM for 16 months had no effect on inflammatory cells.

Myokines, which are produced by repetitive muscle contractions, increase cytotoxicity and play an important role in immune cell infiltration into tumors [11]. Benatti et al. [12] reported that myokines have anti-inflammatory effects. They regulate NK cell proliferation, maturation, and activation, and increase the production of the interleukin (IL)-1 receptor antagonist and IL-10 by blood monocytes. In particular, the amount of myokines released may depend on exercise intensity, contracted muscle mass, and type of exercise performed by an individual [13]. However, several previous studies that administered RE at 80% intensity to FBCSs did not show a positive effect on improving immune and inflammatory cells. This suggests that the RE program should be re-organized with respect to exercise intensity, type, and duration. Therefore, we intended to administer an RE program that could have a positive effect on the improvement of physical strength, immunity, and inflammatory cells in FBCSs, taking into account exercise intensity, amount of exercise, method, and safety.

Circuit resistance exercise is a form of continuous exercise that applies the circuit concept to RE and minimizes the rest period between exercise sets. This exercise method aims to improve physical attributes, such as muscular strength, muscular endurance, cardiorespiratory endurance, and power, through repeated training within a certain period and by prescribing movements that activate several parts of the body [14,15]. The physiological effects of circuit resistance exercise have been verified by several studies [16,17,18,19]. Tabata et al. [20] reported that high-intensity exercise can help to prevent colorectal cancer by enhancing the secretion and elevating the blood concentration of myokines. It is therefore necessary to examine the effect of high-intensity circuit resistance exercise (HCRE) on FBCSs.

The purpose of this study was to examine the effect of HCRE on physical strength, inflammation, and immune cells in FBCSs.

## 2. Materials and Methods

### 2.1. Participants

Thirty female breast cancer survivors volunteered to participate (*n* = 30), with fifteen randomly assigned to the HCRE group (age, 54.7 ± 5.1 years; weight, 58.7 ± 9.4 kg; and height, 1.58 ± 0.46 m) and the other fifteen randomly assigned to the control group (age, 55.4 ± 4.3 years; weight, 59.1 ± 8.1 kg; and height, 1.61 ± 0.71 m). Using G*Power Version 3.1.9.7, we calculated the sample size as 30 subjects, including 15 subjects in each group, which satisfied the effect size of 0.4, error of 0.05, power of 0.80, and an attrition rate of 20%. The purpose and method of the study were explained to the subjects and informed consent form was obtained. The FBCSs were stratified according to age (<50 or >50 years) and current use of hormone therapy (Yes or No). Twenty-one of the subjects had experience with hormone therapy. The criteria for selection of participants were as follows: (a) stage I to IIIA breast cancer with no evidence of recurrence and treatment completed >2 years ago; (b) treatment with surgery, radiation, and chemotherapy; (c) age between 40 and 60 years; (d) physical activity standards: walking less than 60 min 3 times a week, no vigorous exercise (swimming, running, cycling, etc.), no resistance training for 1 year; and (e) no musculoskeletal disorders, comorbidities, or lymphedema. Five participants dropped out of the study (see Figure S1). This study complied with the CONSORT criteria and was registered with the Clinical Research Information Service (registration number, PRE20220127-002).

### 2.2. Measurements

The physical fitness and physical function of breast cancer patients tend to decrease during breast cancer treatment. Decreased physical fitness and physical function are strongly associated with mortality in breast cancer survivors [6,7]. In addition, it has been reported that CRP and NKCA in breast cancer survivors play an important role in the prevention of cancer recurrence [9]. We selected variables that affect the health of breast cancer survivors based on previous studies. Measurements included body composition, physical fitness, high-sensitivity C-reactive protein (hsCRP), and NK cell activity (NKCA), which were performed before and after 12 weeks of exercise, for 2 days at each time points. Body composition measurement and blood sampling were performed on the first day. The participants restricted food intake for 12 h and restricted exercise and drugs that could affect the results for 24 h before blood sampling. Physical fitness (grip strength, back strength, sit-up, plank, sit-and-reach, whole-body reaction, standing on one leg with eyes closed, and Y-balance test) was assessed on the second day. The participants restricted food intake for 2–3 h and exercise for 24 h before physical fitness assessment. Running (10 min) and dynamic stretching (10 min) were performed before the physical fitness tests. Body fat and muscle mass were measured using bioelectrical impedance analysis with a body composition analyzer (Inbody 520, Biospace, Seoul, Korea). For 12 weeks, the HCRE group underwent HCRE for 50 min a day and 2–3 times a week. The control group underwent only activities of daily living during the study period.

#### 2.2.1. Physical Fitness Tests

Five physical factors were measured, namely, muscular strength, muscular endurance, flexibility, reaction time, and balance. The details regarding measurement sequence and method are presented below:

1. Muscular strength was measured using grip and back muscle strength tests. Grip strength was measured using a grip strength measuring instrument (TKK-5401, Takei, Niigata, Japan). During the measurement, the subject stood straight with the arms straightened and placed 20 cm from the body. The dynamometer was then held for measurement at a right angle across the first joint of the finger [21]. Back muscle strength was measured using a back muscle strength measuring instrument (TKK-5402, Takei, Niigata, Japan). The posture assumed by the subject during measurement was to straighten the knees and place both feet in the shape of the number 11. The subject then grabbed the handle attached to the instrument and pulled it vertically by applying force to the entire body. Measurement precautions were fully explained before the muscle strength measurement, and the highest record was adopted by measuring twice [21].
2. Muscular endurance was measured using the sit-up and plank tests. Sit-ups were measured using a sit-up measuring instrument (BS-SU, Inbody, Seoul, Korea) [22,23]. The instrument automatically recorded repetitions when the head was recognized by the sensor attached to the instrument. The number of repetitions performed for 30 s was recorded [24]. Planks were measured using a stopwatch. The subject raised the upper body from the prone position on the mat, using the elbows. Then, the hips and legs were lifted, and the neck, trunk, and lower body were aligned in the sagittal plane. If the body touched the mat or incorrect posture was identified twice, the measurement was stopped and recorded [25].
3. Flexibility was measured using the sit-and-reach test. Sit-and-reach was measured using a sit-and-reach measuring instrument (BS-FF, Inbody, Seoul, Korea) and recorded in units of 0.1 cm. The knee not touching the ground and pushing the measuring plate with the finger were considered fouls, and in such instances, the test was performed again [24].
4. Reaction time was measured using the whole-body reaction test. Whole-body reaction was measured using a whole-body reaction measuring instrument (BS-FF, Inbody, Seoul, Korea). When a mechanical signal sounded, the time taken for the feet to fall off the mat was recorded. The highest record was measured twice and recorded in units of 0.001 s [26].
5. Balance was measured by having the subject stand on one leg with the eyes closed and using the Y-balance test. Standing on one leg with eyes closed was performed with the hands and shoulders parallel and the knee of the non-dominant leg horizontal to the ground. The measurement started from the moment the subject closed their eyes with their arms and legs raised. The Y-balance test required the non-dominant leg to reach as far as possible in three separate directions (anterior, posteromedial, and posterolateral directions) while the dominant leg was balancing. The measurement was performed again if the subject did not return to the initial position or the foot touched the ground during the measurement. Leg length was measured from the anterior superior iliac spine to the medial malleolus. The composite score was calculated using the following formula [27]:

(1) $$Composite\;score=\frac{\left(Anterior+Posteromedial+Posterolateral\;direction\right)}{3\times Right\;limb\;length}\times 100$$

#### 2.2.2. Assessment of Inflammation and Immune Cells

Blood was drawn at 08:00 a.m. the day after maintaining a fasting state for 12 h. Subjects underwent their usual daily activities and sleep 3 days before blood sampling, and avoided activities such as high-intensity exercise and consumption of caffeine and alcohol. Subjects were allowed to rest for 30 min, and then blood was collected from the forearm vein using an injection needle. For the analysis of hsCRP, 9 mL of blood (−70 °C) was placed in a disposable vacutainer tube and stored in a container. For the analysis of NKCA, 1 mL of blood was placed in a dedicated tube using the NK Vue^®^ kit (ATGen Co., Seongnam, Korea).

1. hsCRP was measured by turbidimetric immunoassay using a Cobas 8000 C702 (Roche Diagnostics System, Basel, Switzerland) instrument [28].
2. For NKCA measurement, NK cells from whole blood were artificially activated using an ELx808 reader (BioTek Instruments, Winooski, VT, USA), and then the secreted interferon-γ levels were quantified by enzyme-linked immunosorbent assay [29].

### 2.3. High-Intensity Circuit Resistance Exercise

The exercise program used in this study complied with the guidelines for physical activity for cancer survivors of the US Department of Health and Welfare and the American Society of Sports Medicine [30,31]. The HCRE program for FBCSs was modified and supplemented with the circuit resistance exercise program used in the studies by Lee et al. [32] and Ballor et al. [33]. The HCRE program consisted of warm-up (walking and stretching, 10 min), main exercise (30 min), and cool-down (stretching, 10 min). One set consisted of 8 exercises with a three-minute rest period after each set. Pneumatic exercise equipment (Inbody, Seoul, Korea) was used to ensure the safety of the FBCSs. The exercise intensity was calculated as 7–10 RM in contrast to 1 RM in the indirect estimation method of Brzycki [34]. Participants were educated about exercise motions for 2 days before participating in the HCRE program. The HCRE program was conducted under the supervision of a professional trainer, and the details are presented in Table 1.

### 2.4. Statistical Analysis

The data were processed using Statistical Package for the Social Sciences (version 23.0; SPSS Inc., Chicago, IL, USA). Variables are presented as mean ± standard deviation. The normal distribution of the data was confirmed using the Shapiro–Wilk method. Two-way repeated-measures analysis of the variance was used to examine the interaction effects between groups before and after the HCRE program. The statistical significance level was set at α = 0.05.

## 3. Results

### 3.1. Body Composition

Changes in body composition are presented in Table 2. Body mass index (BMI) (*F* = 8.794, *p* = 0.007), body fat (*F* = 19.291, *p* = 0.001), and muscle mass (*F* = 49.145, *p* < 0.001) showed an interactive effect. In the effect test of the difference between time points, significant differences were found in body fat (*F* = 9.892, *p* = 0.005) and muscle mass (*F* = 5.531, *p* = 0.028), but there was no difference in BMI (*F* = 0.017, *p* = 0.899). In the effect test of the difference between groups, no significant differences were found in BMI (*F* = 0.031, *p* = 0.862), body fat (*F* = 0.542, *p* = 0.539) or muscle mass (*F* = 0.165, *p* = 0.689).

### 3.2. Physical Fitness

Changes in physical fitness are presented in Table 3. Grip strength (*F* = 13.658, *p* = 0.001), back strength (*F* = 7.085, *p* = 0.014), sit-up (*F* = 5.463, *p* = 0.028), reaction time (*F* = 4.579, *p* = 0.043), standing on one leg with eyes closed (*F* = 5.501, *p* = 0.028), and Y-balance test composite score (*F* = 6.013, *p* = 0.022) showed an interactive effect, but plank (*F* = 2.035, *p* = 0.167) and sit and reach (*F* = 2.080, *p* = 0.163) did not show an interactive effect. In the effect test of the difference between time points, significant differences were found in grip strength (*F* = 9.723, *p* = 0.005), sit-up (*F* = 7.296, *p* = 0.013), plank (*F* = 4.360, *p* = 0.048), standing on one leg with eyes closed (*F* = 8.668, *p* = 0.007), and Y-balance test composite score (*F* = 16.116, *p* = 0.001), but there were no differences in back strength (*F* = 2.253, *p* = 0.147), sit and reach (*F* = 4.082, *p* = 0.055), or reaction time (*F* = 3.642, *p* = 0.069). In the effect test of the difference between groups, significant differences were found in reaction time (*F* = 4.579, *p* = 0.043) and Y-balance test composite score (*F* = 4.427, *p* = 0.047), but there were no differences in grip strength (*F* = 0.000, *p* = 0.984), back strength (*F* = 0.725, *p* = 0.403), sit-up (*F* = 0.104, *p* = 0.750), plank (*F* = 0.084, *p* = 0.774), sit and reach (*F* = 0.856, *p* = 0.364), or standing on one leg with eyes closed (*F* = 1.222, *p* = 0.280).

### 3.3. Inflammation and Immune Cells

Changes in inflammation and immune cells are presented in Table 4. NKCA (*F* = 7.540, *p* = 0.012) showed an interactive effect, but hsCRP (*F* = 1.841, *p* = 0.188) did not. In the effect test of the difference between time points, a significant difference was found in NKCA (*F* = 6.815, *p* = 0.016), but there was no difference in hsCRP (*F* = 0.986, *p* = 0.331). In the effect test of the difference between groups, no significant differences were found in hsCRP (*F* = 0.094, *p* = 0.757) or NKCA (*F* = 0.180, *p* = 0.657).

## 4. Discussion

Previous studies have emphasized that various exercise types and physical activities are effective ways to improve the body function of FBCSs. RE contributes to improved physical function and lower cancer recurrence and mortality rates in FBCSs. However, there is no guideline for the use of HCRE to induce improvement in physical strength and immune function in patients with breast cancer. Therefore, we aimed to provide up-to-date information through this study, in order to elucidate the potential management strategies for the physical and immune dysfunctions that occur after breast cancer treatment.

Changes in body weight are a well-known side effect of breast cancer treatment. They are associated with increased fat and decreased muscle mass, while changes in body composition are associated with increased risk of breast cancer recurrence and death [35]. Obesity, characterized by high fat mass and low muscle mass, is one of several potentially modifiable factors that can influence breast cancer. In particular, several studies have provided evidence that postmenopausal obesity is associated with a higher risk of breast cancer-related mortality and recurrence [36]. RE is one of the various exercise methods that can positively change body composition. It is effective in improving muscle mass and has a positive effect on changes in body fat according to the type, intensity, and duration of the exercise. Hagstrom et al. [9] reported that RE for 16 weeks was effective in reducing body fat percentage in FBCSs. Schmitz et al. [37] reported that RE for 6 months decreased body fat, body fat percentage, and waist circumference in FBCSs. In this study, we administered HCRE to FBCSs, and found that HCRE for 12 weeks proved effective in improving their BMI, body fat, and muscle mass. Similar to previous studies, the muscle mass change in the intervention group was the most notable of the body composition changes [38,39,40]. These findings suggest that HCRE effectively improves body fat and muscle mass. In addition, the HCRE of 30 min/day, 2–3 times/week, for 12 weeks can have the same effect as long-term exercise performance despite its rather short duration.

Physical activity is reduced after breast cancer diagnosis, and physical function remains low for more than 5 years after breast cancer is cured [41,42]. In previous studies, early treatment of breast cancer increased survival rate but severely affected quality of life by lowering physical activity and physical fitness levels [43,44,45]. Exercise is a feasible and well-accepted strategy for improving the physical and mental problems associated with FBCSs [46]. RE has been proven to be effective in improving strength, muscular endurance, physical function, and quality of life of FBCSs [47,48]. Winters-Stone et al. [49] reported that RE increased upper and lower body strength in FBCSs over 50 years of age. Battaglini et al. [50] investigated the effects of RE in FBCSs aged 40–70 years and reported that RE increased muscle strength and decreased fatigue after breast cancer treatment (initial stage). In our study, FBCSs underwent HCRE, and the physical fitness before and after the HCRE showed an interaction effect between the time points and groups with respect to grip strength, back muscular strength, whole-body reaction, standing on one leg with eyes closed, and the Y-balance test. The HCRE program in this study improved muscle strength, muscular endurance, reaction time, and balance in FBCSs.

This study evaluated health-related physical fitness in the same way as previous studies and additionally confirmed various physical fitness variables related to functional movements that are necessary for daily life. As a result, we confirmed that HCRE improved the health-related physical fitness of FBCSs and various physical variables, such as plank, standing on one leg with eyes closed, and the Y-balance test.

Decrease in muscle mass and increase in fat mass are physical characteristics of FBCSs. Decreased muscle mass may result in reduced production of IL-15, a myokine that regulates adipose tissue and promotes NK cell development and survival [51]. RE is a major source of myokines, and long-term exercise adaptation can lower basal levels of inflammatory cytokines [52]. However, in previous studies, resistance training showed a positive effect on improving the physical strength and quality of life of FBCSs, but had little effect on immunity and inflammatory cells. Hagstrom et al. [9] reported that RE for 16 weeks at an intensity of 80% of one RM had no effect on NK cell, NKT cell, C-reactive protein, IL-6, and IL-10 levels. Schmidt et al. [53] reported that RE at an intensity of 60–80% of one RM did not improve C-reactive protein, IL-1, and IL-6 levels in FBCSs. In contrast, Oh et al. [54] reported that IL-15 expression increased after acute and chronic resistance training, suggesting that resistance training may have a positive effect on inflammation and immune cells. In particular, to make RE more advantageous to FBCSs, de Jesus Leite et al. [47] suggested that exercise programs administered to FBCSs should focus on exercise intensity, quantity, method, and stability. This study is the first to administer high-intensity circuit-type resistance training to FBCSs, and it proved that HCRE effectively improved NKCA in FBCSs. Exercise-induced myokine levels may vary depending on the duration and intensity of exercise and the type of muscle fiber. Slow-twitch muscle fibers increase IL-15 at low to moderate intensity, whereas high intensity can increase IL-15 in fast-twitch muscle fibers [54]. HCRE increases the levels of myokines (fast-twitch muscle fibers), which are thought to improve NKCA in FBCSs. In light of these points, HCRE is an alternative exercise method that can improve body composition and physical strength; additionally, it can improve immune cell function of FBCSs. Consequently, HCRE can be expected to have a strong impact on mortality, cancer recurrence, and quality of life.

Our study has potential limitations. First, the sample size was small, and there was some resistance to participation in high-intensity RE; thus, it may not be possible to generalize the study results. Second, we were unable to directly identify the exercise-induced myokines that affect the immune cells. It is necessary to identify the direct relationship between exercise-induced myokines and immune cells. For future studies, large community-based trials should be conducted to evaluate the long-term health-related benefits of RE in FBCSs.

## 5. Conclusions

HCRE for 30 min a day, 2–3 times a week, for 12 weeks was found to improve the body composition, physical strength, and immune cells of FBCSs. These findings support the administration of HCRE to FBCSs as a strategy to counteract the negative effects of breast cancer treatment. In particular, HCRE altered exercise-induced myokine release and increased NKCA in the FBCS. Therefore, this study suggests that HCRE intervention is an opportunity to improve health indicators of fitness and immune cells. In addition, the HCRE administered in this study was performed in a gym equipped with pneumatic equipment to ensure safety of the FBCS. The HCRE program appears to be safe for FBCSs, as no lymphedema or musculoskeletal damage occurred during the program.

## Figures and Tables

**Table 1:** High-intensity circuit resistance exercise program.

Time	Type	Intensity	Frequency
Warm-up (10 min)	Walking and Stretching		
Main exercise (30 min)	Leg Press Seated Row Leg Extension Shoulder Press Back Extension Arm Extension Hip Adduction Hip Abduction	Week 1: 16 rep, 3 set 1 RM 40% Week 2: 12 rep, 4 set 1 RM 60% Week 3–12: 8 rep, 4 set 1 RM 80% concentric and eccentric ratio: 1/1 Set rest: 3 min	2~3/week For 12 weeks
Cool-down (10 min)	Stretching		

**Table 2:** Change in body composition.

Variables	Groups	Baseline	Post	*F*		*p*	*ES* (η^2^)
BMI (kg/m^2^)	HCREG	24.2 ± 3.9	23.3 ± 3.26	T	0.017	0.899	0.277
	CG	23.6 ± 3.0	24.4 ± 3.34	G	0.031	0.862	
				T*G	8.794	0.007 **	
Body fat (%)	HCREG	34.9 ± 7.1	33.3 ± 6.62	T	9.892	0.005 **	0.456
	CG	32.1 ± 5.3	32.4 ± 5.23	G	0.542	0.539	
				T*G	19.291	0.001 **	
Muscle mass (kg)	HCREG	20.4 ± 2.6	21.2 ± 2.5	T	5.531	0.028 *	0.682
	CG	21.4 ± 2.1	21.1 ± 2.5	G	0.165	0.689	
				T*G	49.145	<0.001 ***	

Means ± SD: means and standard deviation; BMI: body mass index; HCREG: high-intensity circuit resistance exercise group; CG: control group; T*G indicates an interaction effect and G indicates a main effect of groups, and T indicates a time effect; * *p* < 0.05, ** *p* < 0.01, and *** *p* < 0.001.

**Table 3:** Change in physical fitness.

Variables	Groups	Baseline	Post	*F*		*p*	*ES* (η^2^)
Grip strength (kg)	HCREG	23.1 ± 3.0	25.0 ± 3.1	T	9.723	0.005 **	0.373
	CG	24.3 ± 3.9	24.2 ± 3.5	G	0.000	0.984	
				T*G	13.658	0.001 **	
Back strength (kg)	HCREG	49.7 ± 8.8	52.3 ± 8.4	T	2.253	0.147	0.236
	CG	55.1 ± 13.1	54.4 ± 13.9	G	0.725	0.403	
				T*G	7.085	0.014 **	
Sit-up (rep)	HCREG	6.4 ± 5.2	8.7 ± 5.4	T	7.296	0.013 *	0.192
	CG	6.9 ± 4.1	7.0 ± 4.5	G	0.104	0.750	
				T*G	5.463	0.028 *	
Plank (s)	HCREG	40.5 ± 12.5	46.0 ± 14.8	T	4.360	0.048 *	0.081
	CG	41.4 ± 10.5	42.4 ± 9.7	G	0.084	0.774	
				T*G	2.035	0.167	
Sit and reach (cm)	HCREG	11.5 ± 3.5	13.2 ± 2.3	T	4.082	0.055	0.083
	CG	13. 7 ± 5.4	14.0 ± 4.6	G	0.856	0.364	
				T*G	2.080	0.163	
Reaction time (s)	HCREG	0.56 ± 0.11	0.50 ± 0.010	T	3.642	0.069	0.209
	CG	0.63 ± 0.16	0.64 ± 0.13	G	4.579	0.043 *	
				T*G	6.080	0.022 *	
Standing on one leg with eyes closed (s)	HCREG	7.6 ± 4.8	9.7 ± 3.8	T	8.668	0.007 **	0.193
	CG	6.9 ± 3.5	7.1 ± 2.6	G	1.222	0.280	
				T*G	5.501	0.028 *	
Y-Balance test composite (%)	HCREG	72.8 ± 7.3	92.1 ± 6.4	T	16.116	0.001 **	0.207
	CG	76.1 ± 13.7	80.8 ± 5.5	G	4.427	0.047 *	
				T*G	6.013	0.022 *	

Means ± SD: means and standard deviation; BMI: body mass index; HCREG: high-intensity circuit resistance exercise group; CG: control group; T*G indicates an interaction effect and G indicates a main effect of groups, and T indicates a time effect; * *p* < 0.05 and ** *p* < 0.01.

**Table 4:** Change in inflammation and immune cells.

Variables	Groups	Baseline	Post	*F*		*p*	*ES* (η^2^)
hsCRP (mg/L)	HCREG	0.20 ± 0.14	0.14 ± 0.07	T	0.986	0.331	0.373
	CG	0.18 ± 0.15	0.19 ± 0.12	G	0.094	0.757	
				T*G	1.841	0.188	
NKCA (p g/mg)	HCREG	773.0 ± 668.6	1092.7 ± 816.2	T	6.815	0.016 *	0.236
	CG	809.4 ± 784.6	801.3 ± 786.3	G	0.180	0.675	
				T*G	7.085	0.014 *	

Means ± SD: means and standard deviation; hsCRP: high sensitivity C-reactive protein; NKCA: natural killer cell activity; HCREG: high-intensity circuit resistance exercise group; CG: control group; T*G indicates an interaction effect and G indicates a main effect of groups, and T indicates a time effect; * *p* < 0.05.

## Data Availability

The data presented in this study are available upon request from the corresponding author.

## Supplementary Materials

The following supporting information can be downloaded at: https://www.mdpi.com/article/10.3390/ijerph19095463/s1, Figure S1: Consolidated Standards of Reporting Trials flow diagram.
