# Supplementary material for: Impact of High-Intensity Circuit Resistance Exercise on Physical Fitness, Inflammation, and Immune Cells in Female Breast Cancer Survivors: A Randomized Control Trial
Source: Int J Environ Res Public Health. 2022 Apr 29;19(9):5463. doi: 10.3390/ijerph19095463 (PMC9102474; doi:10.3390/ijerph19095463)
Supplement: Supplementary file 1 [file ijerph-19-05463-s001.zip › ijerph-1634418-supplementary.pdf]

CONSORT

TRANSPARENT REPORTING of TRIALS

CONSORT 2020 Flow Diagram

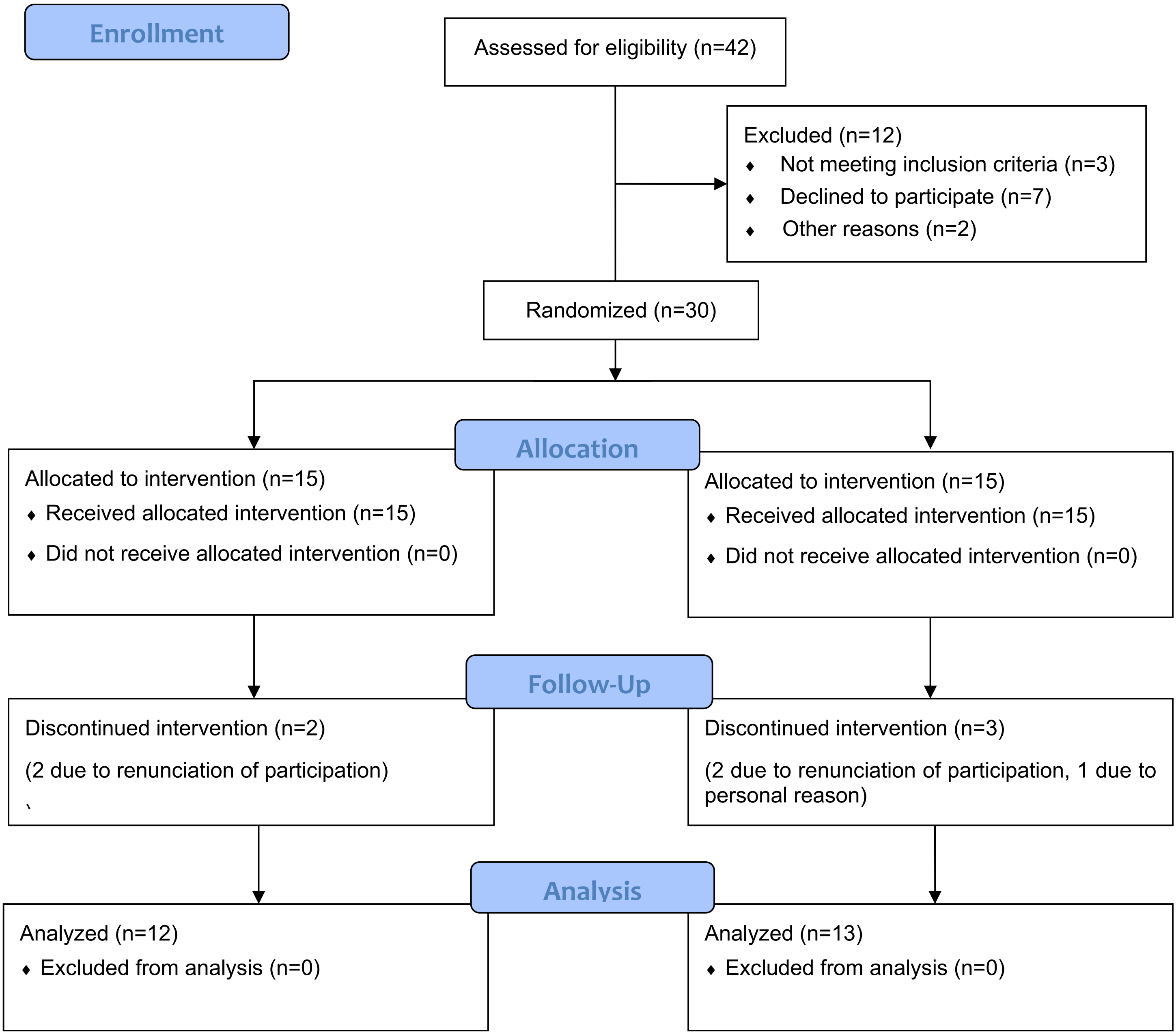

Figure S1. Consolidated Standards of Reporting Trials flow diagram.
